# Is the Mitochondrial Membrane Potential (∆Ψ) Correctly Assessed? Intracellular and Intramitochondrial Modifications of the ∆Ψ Probe, Rhodamine 123

**DOI:** 10.3390/ijms23010482

**Published:** 2022-01-01

**Authors:** Ljubava D. Zorova, Evgeniya A. Demchenko, Galina A. Korshunova, Vadim N. Tashlitsky, Savva D. Zorov, Nadezda V. Andrianova, Vasily A. Popkov, Valentina A. Babenko, Irina B. Pevzner, Denis N. Silachev, Egor Y. Plotnikov, Dmitry B. Zorov

**Affiliations:** 1 A.N. Belozersky Institute of Physico-Chemical Biology, Lomonosov Moscow State University, 119992 Moscow, Russia; korsh@genebee.msu.ru (G.A.K.); tashlitsky@belozersky.msu.ru (V.N.T.); zorov@inbox.ru (S.D.Z.); andrianova@belozersky.msu.ru (N.V.A.); popkov.vas@gmail.com (V.A.P.); nucleus-90@yandex.ru (V.A.B.); irinapevzner@mail.ru (I.B.P.); proteins@mail.ru (D.N.S.); plotnikov@belozersky.msu.ru (E.Y.P.); 2 Biological Faculty, Lomonosov Moscow State University, 119992 Moscow, Russia; demchenko.jane@mail.ru; 3 Faculty of Bioengineering and Bioinformatics, Lomonosov Moscow State University, 119992 Moscow

**Keywords:** mitochondria, energetics, membrane potential, fluorescence, cytochrome P450, esterase, tumor cells, cancer

## Abstract

The mitochondrial membrane potential (∆Ψ) is the driving force providing the electrical component of the total transmembrane potential of hydrogen ions generated by proton pumps, which is utilized by the ATP synthase. The role of ∆Ψ is not limited to its role in bioenergetics since it takes part in other important intracellular processes, which leads to the mandatory requirement of the homeostasis of ∆Ψ. Conventionally, ∆Ψ in living cells is estimated by the fluorescence of probes such as rhodamine 123, tetramethylrodamine, etc. However, when assessing the fluorescence, the possibility of the intracellular/intramitochondrial modification of the rhodamine molecule is not taken into account. Such changes were revealed in this work, in which a comparison of normal (astrocytic) and tumor (glioma) cells was conducted. Fluorescent microscopy, flow cytometry, and mass spectrometry revealed significant modifications of rhodamine molecules developing over time, which were prevented by amiodarone apparently due to blocking the release of xenobiotics from the cell and their transformation with the participation of cytochrome P450. Obviously, an important role in these processes is played by the increased retention of rhodamines in tumor cells. Our data require careful evaluation of mitochondrial ∆Ψ potential based on the assessment of the fluorescence of the mitochondrial probe.

## 1. Introduction

The mitochondrial membrane potential (∆ψ) is a highly important part of the mitochondrial existence and functioning [1]. For a long time, it was assumed that the only function of ∆ψ was participation in oxidative phosphorylation as an intermediate in the process of energy storage in the coupling membranes, including the inner membrane of mitochondria [2,3], as part of the proton-motive force driving the rotary part of the ATP synthase. However, by the end of the XX century, a separate, then not yet understood role in alternative mitochondrial functions was assigned to ∆ψ [4]. This was based on the fact that the membrane potential was maintained by the reversal of the ATP synthase reaction under conditions when a normal generation of ∆ψ is impossible (for example, in anoxia or metabolic inhibition, when the mitochondrial respiratory chain becomes inactive and incapable of electrogenesis) [5]. This indicated an extremely important role of ∆ψ in the functioning of mitochondria, and maybe the whole cell in energy-unrelated processes. In turn, this explained the unusually high intracellular concentration of ATP, which was orders of magnitude higher than the affinity of ATP-consuming intracellular enzymes, thus indicating the buffering role of ATP to maintain ∆ψ in critical situations.

Earlier, we have overviewed known options of utilizing ∆ψ for non-energetic needs of mitochondria and cells [1]. Among them, we found important the provision of bidirectional transport of charged constituents including ions, proteins and nucleic acids. In addition, in recent years, it has become clear that in the mitochondrial quality control system, ∆ψ plays the pivotal role of a critical regulator, based on the assessment of which decision is made: to preserve the mitochondrion, or to dispose it in the process of auto/mitophagy [6,7] when the values of ∆ψ substantially drop to a certain limit, reporting on a decrease in its functionality. Thus, the urgent need for the cellular homeostasis of ∆ψ becomes obvious [1], due to the fact that its long-term reduction to critical levels entails not only the death of the mitochondria, but also of the cell as a whole.

In 1982, the study made in Lan Bo Chen’s group established a new background for the development of the mitochondrial approach in anti-cancer strategy [8]. The remarkable ability of tumor cells to accumulate and retain probes for mitochondrial ∆ψ provided a long-awaited approach to distinguish between normal and tumor cells and to use this ability in cancer therapy. Further work from this group interpreted these unique properties of tumor cells as a reflection of higher values of cellular and mitochondrial membrane potentials in cancer cells compared to non-tumor cells [9,10,11]. However, this difference was too small to provide such a significant difference in the retention of the fluorescent probe in two types of cells.

On the other hand, the detailed analysis of this data has called into question not only the interpretation of the data but also the adequacy of determination of mitochondrial membrane potential in cells of various types by the ability to distribute the potential-sensitive probe truly reflecting the value of the membrane potential [1,12]. This is especially true for fluorescent probes of ∆ψ, especially rhodamine 123 (Rh123) or esters of tetramethylrhodamine (TMRM or TMRE), whose fluorescence intensity in mitochondrial matrix in a big range of their concentrations become higher with an increase of the membrane potential.

It should be noted that fluorescent probes for mitochondrial ∆ψ have become the main tool for evaluating the functional activity of mitochondria in cells [13]. Theoretically, the accumulation of permeable charged (positive in case of mitochondria) fluorescent probes that is driven by the electric component of the transmembrane potential of hydrogen ions follows the Nernst equation,

(1) Δψm = −(RT/nF) log(Fin/Fout)

Hence, the potential difference on the inner mitochondrial membrane of 59 mV (minus inside) determines a ten-fold accumulation (F*_in_*/F*_out_* = 10) of the fluorescent cationic probe in the mitochondrial matrix compared to the extra-mitochondrial volume. However, the discrepancy between theory and practice and the ambiguous interpretation of changes in the probe fluorescence in response to a challenge when working with cells is caused by a set of possibilities that we had considered earlier. They are determined by the following factors:

1. Binding of the fluorescent probe to cellular elements and structures of the mitochondrial matrix. Theoretically, positively charged hydrophobic/amphiphilic drugs can be bound to intracellular anionic constituents such as phospholipids and nucleic acids. In addition, the electrostatic interaction of cationic probes with the anionic moiety of proteins must also be considered and among those, mitochondrial proteins, so-called chargerins were proposed to play a role as binding partners of mitochondrial probes [14,15,16].
2. Transformation of the permeable form of the probe into an impermeable form with the preservation of fluorescent properties. For example, as a result of deesterification by intracellular and intramitochondrial esterases, the highly membrane-permeable Rh123 (as well as tetramethyl rhodamines, TMRM, or TMRE) was proposed to be converted into the corresponding zwitterion (rhodamine 110, Rh110), in contrast to the parental cationic form, having a very low membrane permeability [17] thus possibly entrapping the modified probe in the compartment where the modification occurred.
3. Intracellular/intramitochondrial degradation of the probe. We admit that not much data exists on this issue, but all probes introduced into the cell can be considered as xenobiotics, which obey the common cellular rule to be expelled from the cell either in intact form or after its modification, often with the involvement of cytochrome P450 [18,19]. There were attempts to make minimal analysis of the components being a result of catabolism of Rh123, but the exact identification of those has not been done [20,21].
4. The change in the contribution of the membrane potential on the cell membrane to the total fluorescence of the probe in the mitochondrial matrix [12].
5. Changes in the values of ∆Ψ on the inner mitochondrial membrane due to the possible transformation of membrane potential to the concentration difference of pH values (∆pH) while maintaining the values of the transmembrane potential of hydrogen ions (∆µH^+^) [2,8].
6. Activity of non-specific pumps in the cell and mitochondrial membrane efflux of xenobiotics in cells and mitochondria. The protein-meditated transport of xenobiotics (including mitochondrial cationic probes) across membranes is predominantly executed by the solute carrier (SLC) and ATP-binding cassette (ABC) superfamilies of proteins [22,23,24]. While SLC family members are responsible for both inward and outward transport, ABC transporters exclusively provide efflux mechanism in an energy-dependent fashion to overcome a concentration gradient. Of the latter, one of the most discussed mechanisms for expelling cationic dyes out of the cell is that they are very good substrates for a multidrug resistance protein (MDR) [25] highly involved in the occurrence of insensitivity of cells to a pharmacological therapy. Similar mechanisms of expelling drugs from mitochondria have been reported [26,27].

The assessment of the contribution of each of these factors is very complex, but it becomes critical for the accurate compliance of changes in the fluorescence of the probe with real membrane potential. Realizing that an intracellular chemical modification of the probe to the membrane potential (Rh123 or TMRM and TMRE) can lead to a false interpretation of the values of fluorescence as an index of the membrane potential, the purpose of this study is to explore the possibility of such modifications for Rh123.

## 2. Results

**Figure 1:**
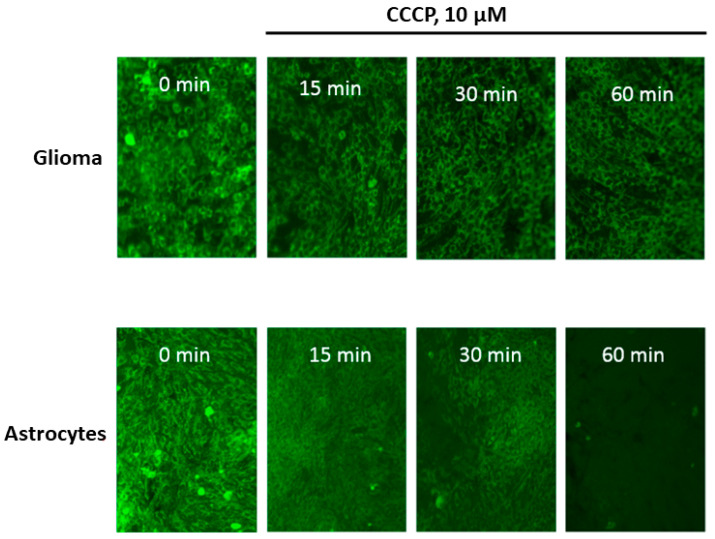
The time-dependent loss of fluorescence of Rh123 caused by the uncoupler in cells attached to the substrate (glioma C6, upper row and astrocytes, lower row). The cells were incubated for 2 h with Rh123 (2 µM), after which they were placed in a rhodamine-free medium containing 10 µM of CCCP, and fluorescence was recorded in time intervals (15, 30, and 60 min) using conventional fluorescent microscopy. Before taking the picture, at each time, we placed the cells in a rhodamine-free culture medium. The images represent the near monolayer state of cell cultures with approximately 200 cells in the field of view.

At the first step of the study, we compared the degree of release of Rh123 from glioma cells and astrocytes after the elimination of the proton-motive force by adding an uncoupler CCCP in concentrations that deliberately eliminate the electrochemical potential of hydrogen ions in mitochondria (10 µM). The fundamental difference from the conditions for the release of Rh123 from mitochondria and cells observed in [8] consisted in the fact that in this work, the mitochondria in the cells retained the membrane potential and the release was carried out due to the natural leakage of Rh123 from the cells into an environment in which Rh123 was absent, while in our conditions, the basis for the release of Rh123 was the elimination of the driving force of the accumulation of Rh123 in the cell. From Figure 1 it can be seen that there is a delayed release of Rh123 in glioma cells, while after 60 min of exposure to the CCCP, almost no Rh123 remains in astrocytes, in glioma cells, the fluorescence is present at a fairly high level. After 2 h, glioma cells dramatically lose the fluorescence and after 18 h almost no fluorescence can be detected in glioma cells (not shown). Our analysis showed that there is no significant difference between the content of mitochondria in the two types of cells. We also note the initially increased heterogeneity of fluorescence of Rh123-stained astrocytes compared to glioma cells, which demonstrate a fairly homogeneous distribution of fluorescence between cells.

**Figure 2:**
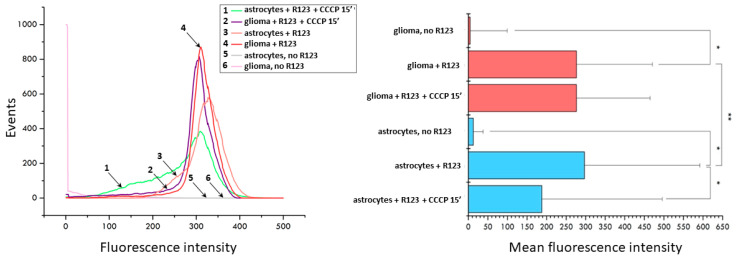
Uncoupler-induced changes in fluorescence of Rh123-stained astrocytes and glioma cells evaluated by flow cytometry. Left panel, distribution of fluorescence (1, green, astrocytes incubated with 2 µM Rh123 and 10 µM CCCP; 2, purple, glioma cells incubated with Rh123 and CCCP for 15 min; 3, pink, as in 1 but without CCCP; 4, red, as in 2 but without CCCP; 5, grey, astrocytes, no additions; 6, light pink glioma, no additions). Right panel, mean fluorescence of Rh123 based on data showing in left panel. * *p* < 0.05 according to the Wilcoxon criterion, ** *p* < 0.05 according to the Mann-Whitney U-test.

The fluorescence microscopy data was confirmed by flow cytometry. A brief analysis of the populations of glioma cells and astrocytes stained with Rh123 and subjected to 15 min treatment of the CCCP shows that initially the population of astrocytes is heterogeneous in terms of the membrane potential of mitochondria with the presence of a small shoulder on the histogram of distribution, while the histogram of fluorescence of glioma cells shows homogeneous fluorescence with a peak in the region of high values (Figure 2). A 15-min uncoupling results in a sharp increase in low-potential mitochondria in astrocytes and a slight shift of median values to the region of low fluorescence intensity in cells. The same exposure of glioma cells led to a slight decrease in the median values of the fluorescence. This suggests that astrocytes lose positively charged Rh123 molecules when the mitochondrial membrane potential is eliminated significantly faster than glioma cells.

**Figure 3:**
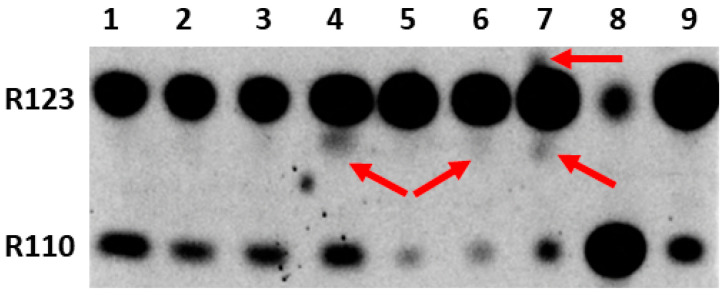
Thin-layer chromatogram of butanol extracts from rhodamine 123-treated astrocytes and glioma cells and their mitochondria. 1st lane, mitochondria isolated from astrocytes incubated for 2 h with Rh123 (2 µM); 2, mitochondria isolated from intact astrocytes with subsequent 2-h incubation with Rh123 (2 µM); 3, cytoplasmic fraction of astrocytes incubated for 2 h with Rh123; 4, cytoplasmic fraction of glioma cells incubated for 2 h with Rh123 (2 µM), 5, membrane fraction of glioma cells incubated for 2 h with Rh123; 6, mitochondria isolated from glioma cells incubated for 2 h with Rh123 (2 µM); 7, mitochondria isolated from intact glioma cells followed by incubation for 2 h with Rh123 (2 µM); 8, commercial sample of Rh110; 9, commercial sample of Rh 123. Densitometric analysis gave a ratio of Rh123:Rh110 as 51, 47, 35, 33, 15, 18, 15, 24, and 20 for lanes 1–9 correspondingly.

Since our main goal was to determine the possibility and location of the formation of modified forms of Rh123 when comparing cultured normal astrocytes and the C6 glioma tumor cells, butanol extracts from the cells and isolated mitochondria were analyzed by the express thin layer chromatography (TLC). The cells were preincubated for two hours with Rh123 (2 µM). This concentration is slightly higher than the concentrations of Rh123 used for imaging mitochondria in the cell. However, by using these concentrations it was easier to detect low levels of modified products by TLC and mass spectrometry, and additionally, the different toxicities of Rh123 were assessed in different cell types, and the contribution of dye release from the cell by non-specific pumps was insignificant. The separation into fractions was carried out in order to determine the localization of modifications, if any occurred. In an attempt to directly assess the role of mitochondria in the modification of rhodamine molecules, mitochondria were isolated from cells, which were incubated with a probe in a buffer solution in the presence of oxidative substrates. In another set of experiments, mitochondria were isolated from intact culture and separately incubated for two hours with Rh123. From the comparison of lanes 1 and 6 and simultaneously lanes 2 and 7 in Figure 3, it follows that the mitochondria of astrocytes produce a significant level of rhodamine 110 regardless of whether they were in cells exposed to Rh123 or were initially isolated from intact cells, and subsequently exposed to Rh123.

During the mitochondria isolation procedure, we also analyzed intermediate fractions, namely, butanol extract, which was obtained from the pellet obtained after cell homogenization and centrifugation at 1200g (called the membrane fraction). This fraction contains intact cells, nuclei, and plasma membrane fragments. The resulting supernatant (called the cytoplasmic fraction) contains mitochondria and other miniature components of the cytoplasm. A comparison of cytoplasmic fractions from astrocytes and glioma cells (lanes 3 and 4) shows a high level of Rh110 in both extracts, apparently due to its presence in the incubation medium as an impurity in the Rh123 samples. However, a significant difference was found in the presence in cytoplasmic glioma samples of products other than Rh123 and Rh110 (shown by the arrow), which was completely absent in the astrocyte samples. Another comparison of lanes 1 and 6 and separately 2 and 7 in Figure 3 indicates that the modification of rhodamine occurs in the mitochondria of glioma, especially noticeable with a direct exposure of rhodamine 123 to intact mitochondria (indicated by arrows). These modifications are invisible in the samples of astrocytic mitochondria. It cannot be excluded that these products are derivatives of Rh110 formed as a result of the deesterification of Rh123.

**Figure 4:**
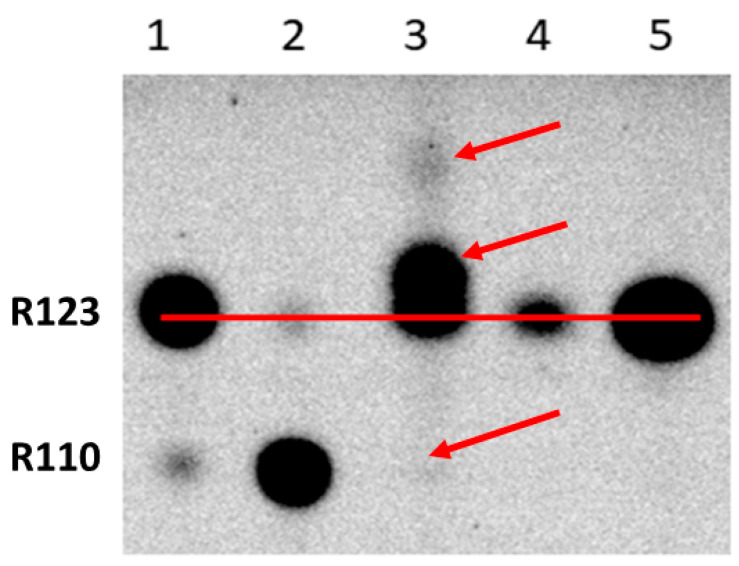
Thin-layer chromatography of butanol extracts of glioma cells and astrocytes treated with Rh123 followed by a long wash. 1st lane, commercial sample of rhodamine 123; 2, commercial sample of rhodamine 110; 3, glioma cells incubated for 2 h with Rh123 (2 µM) and then in pure cultivation medium pure for 22 h; 4, astrocytes incubated 2 h with Rh123 and then for 22 h in pure incubation medium; 5, glioma cells incubated for 2 h with Rh123 (2 µM) and amiodarone (2 µM), and then in pure incubation medium for 22 h. Two arrows at the top indicate modified fluorescent products. For a relative assessment of the mobility of the spots, a horizontal line is drawn along the centers of the spots corresponding to Rh123. The arrow at the bottom indicates the absence of the spot corresponding to Rh110.

Given that glioma cells acquire an enhanced retention of Rh123, in order to explore the dependence of modifications on time, we incubated cells with Rh123 for 2 h and placed them for 22 h in a Rh123-free medium. We found that in glioma cells, after 24 h of cultivation, essential levels of fluorescent products different from Rh123 and Rh110 were observed (see lane 3 of Figure 4). On the other hand, almost no Rh110 was detected in glioma samples, supporting the idea expressed above that modification may originate from Rh110 formed from Rh123 through deesterification. The alternative explanation of the absence of Rh110 in glioma cells after a long period of cultivation is that it is spontaneously expelled from the cells. On the other hand, astrocytes being exposed to similar conditions (2 h of incubation with Rh123 followed by 22-h cultivation in Rh123-free medium) contain very little amounts of Rh123, without even mentioning Rh110 (lane 4). The critical point of these experiments was in that all observed modifications of Rh123 vanished if the incubation with Rh123 was performed in the presence of amiodarone, which can both block the non-specific pump (P-glycoprotein) and inhibit P450-mediated modification (compare lanes 3 and 5) [28].

**Figure 5:**
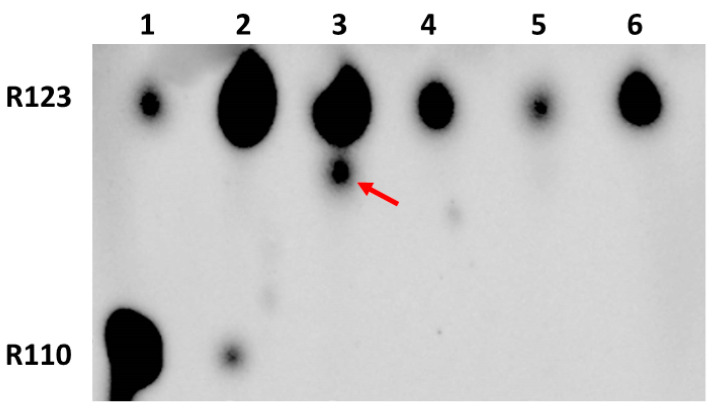
Thin-layer chromatography of butanol extracts of glioma cells and astrocytes exposed to rhodamine 123 and amiodarone. 1st lane, commercial sample of Rh110; 2, commercial sample of Rh123; 3, glioma cells incubated for 2 h with Rh123 (2 µM); 4, glioma cells incubated for 2 h with Rh123 (2 µM) and amiodarone (2 µM); 5, astrocytes incubated for 2 h with Rh123 (2 µM); 6, astrocytes incubated for 2 h with Rh123 (2 µM) and amiodarone (2 µM).

Further use of amiodarone on two types of cells confirmed that even short-term incubation of glioma cells with Rh123 and amiodarone eliminates modifications of rhodamine (lanes 3 and 4 in Figure 5), while similar incubation of astrocytes initially did not lead to chemical modification, and amiodarone in an understandable way increased the content of Rh123 in cells apparently due to the inhibition of the controlled release of Rh123 from cells. The mechanism of amiodarone-induced reduction of Rh123 content in glioma cells remains unclear, but most importantly, the main goal of this study was reached, namely to evaluate the opportunity of modification of the rhodamine molecule, possibly contributing to the obtained values of the mitochondrial membrane potential based on the assumption that all fluorescence in cells belongs to Rh123, since we proved that there are modifications.

**Figure 6:**
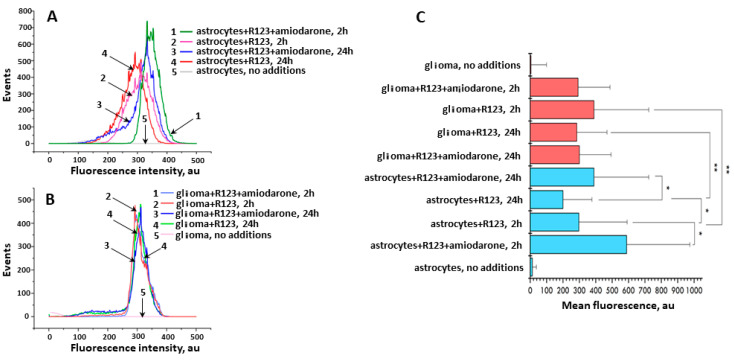
The mean fluorescence intensity measured by flow cytometry in astrocytes and glioma cells incubated with Rh123 (2 µM). (**A**) Distribution of fluorescence in astrocyte cells depending on the agents added to the incubation medium and incubation time; 1, green, astrocytes with Rh123 and amiodarone (2 µM) incubated for 2 h; 2, pink, astrocytes incubated with Rh123 for 2 h; 3, blue, as in 1 but incubated for 24 h; 4, red as in 2 but incubated for 24 h; 5, astrocytes, no additions; (**B**) Distribution of fluorescence in glioma cells depending on the added agents and incubation time; 1, light blue, glioma cells incubated with rhodamine 123 and amiodarone for 2 h; 2, red, as in 1 but without amiodarone; 3, dark blue, as in 1 but incubated for 24 h; 4, green, as in 2 but incubated for 24 h; 5, pink, glioma cell, no additions; (**C**) Median fluorescence of Rh123 in astrocyte and glioma cells treated with Rh123 and amiodarone (2 µM) for 2 or 24 h. * *p* < 0.05 according to the Wilcoxon criterion, ** *p* < 0.05 according to the Mann-Whitney U-test.

In addition, to quantify the intensity of Rh123 fluorescence in two types of cells with further evaluation of the effect of amiodarone on it, under short- and long-term incubation times, we applied flow cytometry, the results of which are shown in Figure 6. While the fluorescence levels in Rh123-loaded glioma cells were rather stable slightly depending on the incubation times and presence of amiodarone, the same conditions for astrocytes caused detectible and statistically significant changes in the total fluorescence of rhodamines. Basically, the flow cytometry data confirm the TLC data, especially if it concerns the median fluorescence values of cells loaded with Rh123. However, a more thorough analysis shows a significantly greater heterogeneity of fluorescence in the astrocyte population, manifested in a wider distribution of fluorescence intensity, while in glioma cells the heterogeneity is less pronounced. If we interpret fluorescence as a reflection of the values of the membrane potential (mainly mitochondrial and to a lesser extent cellular), we can conclude that the values of the mitochondrial membrane potential in glioma is more homogeneous than in astrocytes. Moreover, prolonged incubation of cells with amiodarone loaded with Rh123 led to the appearance of a population of cells with low fluorescence (curve three for astrocytes and curve three for glioma). Note that the same long-term incubation of glioma cells without amiodarone is also accompanied by the presence of a small population with low fluorescence intensity (visible as a small shoulder in curve four for glioma cells). This confirms the data on the selective toxicity of Rh123 for tumor cells initially discovered in the laboratory of Lan Bo Chen [29,30,31,32], thus placing mitochondria as a target in cancer therapy using mitochondria-targeted drugs including fluorescent dyes such as Rh123 [33,34,35,36,37]. It may partially reflect the phenomenon of the extended retention of mitochondrial dyes in tumor cells [8,9,10].

Since thin-layer chromatography is not a very sensitive method, in the next step we switched to using mass spectrometry to detect modified forms of rhodamines, estimated by their molecular mass.

**Figure 7:**
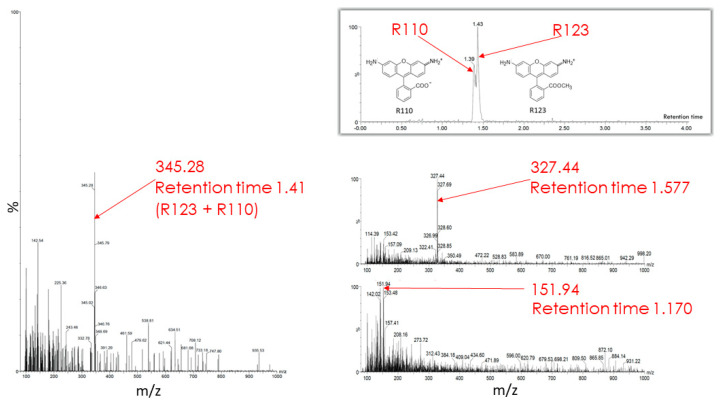
Mass spectrometry of butanol extracts of glioma cells after their cultivation with Rh123 (2 µM) for 2 h. The two factions are resolved with retention times on the column of 1.39 and 1.43 min and a clear predominance of the second component corresponding to Rh123 over the first one corresponding to Rh110 (shown on left spectrum with the mass/charge of 345.28 corresponding to the mixture of Rh123 and 110). Two minor components were detected with retention times of 1.577 and 1.170 min with the main peaks in of mass/charge (*m*/*z*) values of 327 and 152, respectively (two spectra on the right).

**Figure 8:**
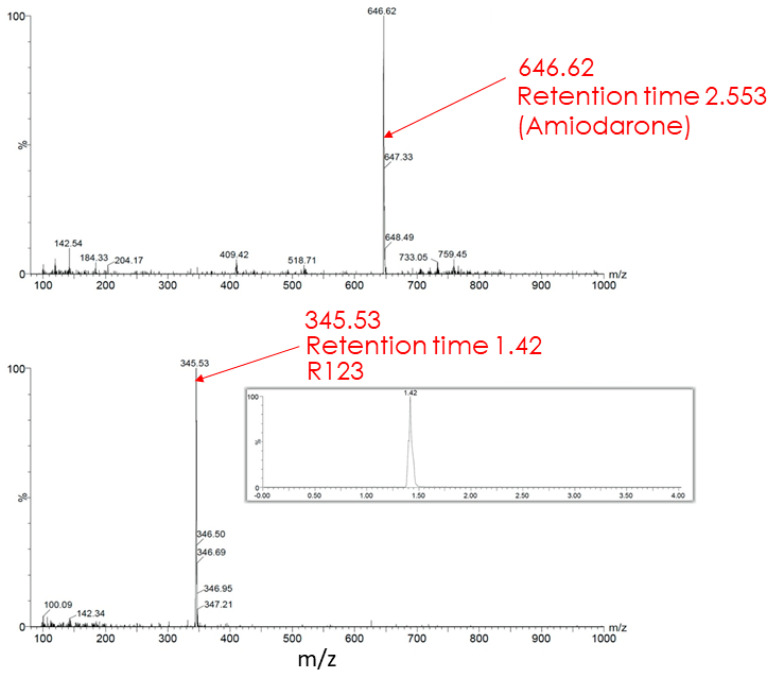
Mass spectrometry of butanol extracts of glioma cells after cultivation with Rh123 (2 µM) and amiodarone (2 µM). Only Rh123 + 110 and amiodarone molecules are detected (with retention times of 1.42 and 2.553 and mass/charge of 345.53 (bottom) and 646.62 (top) correspondingly).

The butanol extract from glioma cells incubated for 2 h with Rh123 contains a number of substances (see description in the Figure 7 legend). Two major fractions were resolved with retention times on the column of 1.39 and 1.43 min (corresponding to Rh110 and Rh123) with a clear predominance of Rh123 over Rh110 in the extract. The major peak had a mass/charge (*m*/*z*) value of 345.28 corresponding to a mixture of Rh123 and 110. In addition, two more minor peaks were resolved with molecular masses of 327.44 and 151.94. The presence of amiodarone in the medium led to abrupt changes in the mass spectrum (Figure 8). As a result of this action, only two peaks corresponding to Rh123 (with mol mass of 345.53) and amiodarone (with mol mass of 646.62) are observed in the butanol extract from glioma cells. This indicates that amiodarone prevents the metabolism of Rh123 in glioma cells.

**Figure 9:**
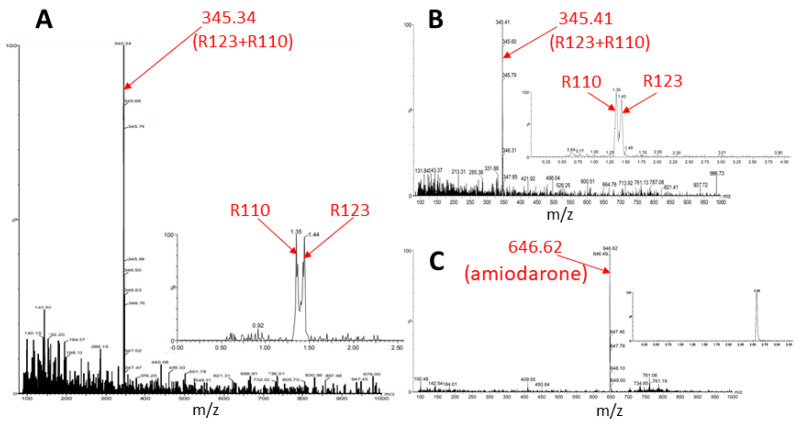
Mass spectrometry of butanol extracts of astroglial cells after cultivation with Rh123 (2 µM, (**A**)) or together with amiodarone (2 µM, (**B**,**C**)). In A, one major component is resolved apparently belonging to rhodamines 123 and 110 with mass/charge = 345.34 with retention times for Rh110 and 123, 1.35, and 1.44 correspondingly (inset). The incubation with amiodarone results in not many changes except the presence of amiodarone (inset).

Similar results were obtained after the incubation of astroglial cells with Rh123 with and without amiodarone. In the absence of amiodarone, after a 2-h incubation of cells with Rh123, no components other than Rh123 and Rh110 were observed, and the presence of amiodarone in the medium did not change the picture—after incubation with amiodarone, only a mixture of Rh123 with Rh110 and amiodarone were detected in butanol extracts of astrocytes (Figure 9). This confirmed the TLC data that astrocytic cells do not have the ability to modify the rhodamine molecule, except for the ability to form Rh110 from Rh123.

**Figure 10:**
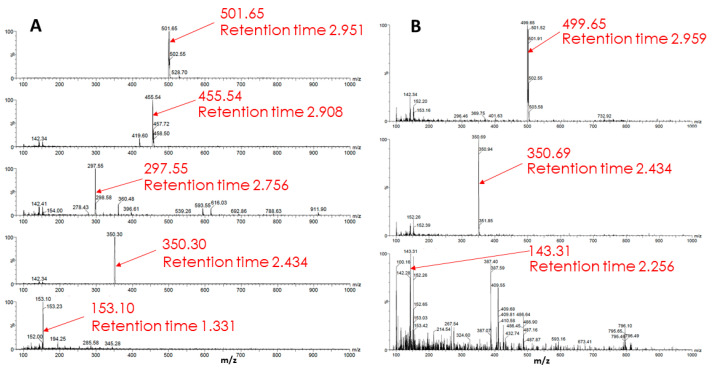
Mass spectrometry of metabolism products of Rh123 (**A**) and Rh110 (**B**) in glioma cells. In A, cells were cultured with rhodamine 123 (2 µM) for 2 h followed by a cultivation in rhodamine-free medium for 22 h. In B, cells were cultivated with 2 µM rhodamine 110 for 2 h.

An analysis of a sample from glioma cells, which, after a standard 2-h incubation with Rh123 incubated in a cultivation medium for an additional 22 h, showed the presence of five components, presumably derivatives of Rh123 (Figure 10). It is noteworthy that the identified substances do not match those found in the extract from glioma cells incubated with Rh123 for 2 h. This implies that further modifications of the substances derived from Rh123 detected in samples after 2 h of exposure occur. To test this assumption, another sample was prepared, which was an extract from glioma cells incubated with Rh110 (2 µM) for two hours. Mass spectrometric analysis revealed three substances in the sample different from the original Rh110. It is worth noting that two of the three components (retention time ~2.95 min, the predominant ion with an *m*/*z* value equal to 501; retention time ~2.43 min, the predominant ion with an *m*/*z* value equal to 350) coincide with the components found in extracts from glioma cells, which, after a standard 2 h incubation with the Rh123 probe, were additionally incubated in pure medium for 22 h. This experimental observation supports the formation of Rh123 modifications in glioma through the step of deesterification and the formation of Rh110.

## 3. Discussion

The direct and main, though not the only, aim of this work was to evaluate methods of measuring the membrane potential using fluorescent dyes, which are positively charged fluorescent molecules in which the positive charge is delocalized by a system of conjugated double bonds, as a result of which, it becomes permeable to the inner membrane of mitochondria, in which the fluorescent cation accumulates in accordance with the values of the membrane potential. The process of cation accumulation in mitochondria stops when the steady state level of cation distribution on both sides of the inner membrane (the mitochondrial matrix is negatively charged) theoretically corresponds to the membrane potential generated by mitochondrial proton pumps. The ratio of the cation in the intermembrane space with respect to the matrix is determined by the Nernst equation given in the Introduction. According to the theory, for every 59 mV, one order of magnitude of the concentrations of the penetrating cation accumulates in the matrix, and if the membrane potential is 177 mV, then the concentration of the cation in the mitochondrial matrix will be 1000 times higher in relation to cytoplasmic levels. However, all this is true if we are dealing with a single molecule, as the law of distribution applies only to this situation. If a molecule is modified in the mitochondrial matrix, and the modified species retains the original fluorescent characteristics, then the membrane potential will continue to drive the initial cation in, as a result of which an additional number of fluorescent molecules will appear in the matrix, exceeding that which should be there in accordance with the theory. By itself, the process of an exceedingly long accumulation of fluorescent cation in cells, which almost does not reach a steady state, should already alert the researcher.

Theoretically, it is possible to search for potential candidates for the presented modifications based on the obtained molecular weights. However, a huge number of variants, in particular with there being possible products of multiple cytochrome P450 activities, including oxidation, reduction, desaturation, ester cleavage, ring expansion, ring formation, aldehyde scission, dehydration, etc. [38] make these attempts quite non-productive. We found that, as a result of modifications of the rhodamine molecule, fluorescent derivatives arise not only with a lower molecular mass, but also having significantly higher values than the parent molecule, which is not surprising, given the previously discovered conjugation of Rh123 with glucuronic acid [20]. Of course, assuming some toxicity of rhodamine dyes for tumor cells, we cannot assess the individual toxicity of each of the formed modification products of the original Rh123 molecule, as that is beyond the scope of this study. However, it is the observed long-term retention of Rh123 (and maybe its products) that may be responsible for the suppression of mitochondrial functions [39] and ultimately for cell death. The mechanisms of the anchoring of rhodamine dyes in tumor cells and their mitochondria remain unclear and their elucidation will not only help solve the basic problems, but can also apply these results in clinical practice.

## 4. Materials and Methods

### 4.1. Cell Cultures

Two cell cultures were used in the study. The culture of rat astroglial cells was obtained with the modified method of McCarthy and de Vellis [40] from the brain of two-to three-day-old rats. Experiments were carried out using outbred animals kept in a vivarium on a standard diet. The experiments were approved by the Bioethics Committee of the A.N.Belozersky Research Institute of Physico-Chemical Biology of Moscow State University. A commercial C6 glioma cell line from the collection of cell lines of the Belozersky Institute was used as the second cell culture. Cells were cultivated in the complete medium containing DMEM/F-12 (1:1), 10% of a fetal bovine (PAA Laboratories, USA) supplemented with 1% glutamine, 1% vitamins (Paneco, Russia), and 2% amino acids (Gibco, Waltham, MA, USA).

### 4.2. The Isolation of Mitochondria

Mitochondria were isolated from cell cultures with the standard method of differential centrifugation. For this purpose, cells dissociated from cultural flasks by 2 mL of trypsin were centrifuged in an Eppendorf 5702 centrifuge (5 min, 600 g), and the precipitate was diluted in 2 mL of cooled non-ionic medium for mitochondrial isolation (300 mM mannitol, 0.5 mM EGTA, 20 µM Hepes, pH 7.4). Next, the cells were homogenized and sequentially centrifuged at 1200 and 8000× *g* (2 °C), respectively.

### 4.3. Thin-Layer Chromatography

Butanol extracts were obtained by adding 96% butanol to the vial containing a pellet obtained after centrifugation in an equal volume or a supernatant in the ratio of 1 volume of butanol to 5 volumes of the supernatant. After gently shaking for 2 min, the system was left for the time necessary for the formation of the butanol fraction at the top of the vial. Butanol extracts were removed from the vial, evaporated in a rotary evaporator RV 3 Flex at a temperature of 40°C and rotation mode 5.5 until the liquid was completely evaporated. The dry residue was dissolved in 50% ethanol. The resulting solution was analyzed by thin-layer chromatography on silica gel plates (TLC Silica gel 60 F254 (Merck, Darmstadt, Germany)). As a mobile phase, a mixture of butanol–water–ethanol was selected in a ratio of 9: 2: 1. Initially, samples with a volume of 1 µL were deposited at a level of 1 cm from the lower edge of the plate, which was immersed in a solvent system. Furthermore, in some cases, the application was normalized; for each sample, such a volume of application was selected in order to unify the detected area of the component corresponding to Rh123. This allowed us to estimate the relative contribution of other detectable components. Next, the membranes were dried and photographed on a ChemiDoc MP device with proper software. To detect Rh123, fluorescence was excited at a wavelength of 488 nm and collected emission at >530 nm.

### 4.4. Fluorescent Microscopy

The study assessed the fluorescence levels of various fluorescent mitochondrial probes, as well as the morphological structure of the mitochondrial network of astrocyte cells and glioma C6 under the influence of various agents. When using the Axiovert conventional fluorescence microscope (Carl Zeiss, Oberkochen, Germany), visualization was performed using ventilated cultural flasks (Corning, New York, NY, USA), and the cells were previously plated onto glass-bottom Petri dishes. Cells were grown in a CO_2_ incubator at 37 °C and 5% CO_2_. Before confocal microscopy, the cells were placed in a bicarbonate-free medium buffered with Hepes.

Each sample was imaged 3 times on a fluorescence microscope, whereas with confocal scanning, each sample was imaged 4 times. The final images obtained from the confocal microscope are the result of averaging four scans. The scanning speed, signal amplification level, and resolution of the resulting image were the same for all experiments in each series. The thickness of the confocal plane was set at 5 microns (pinhole 150). To excite the fluorescence, an argon laser with excitation at 488 nm was used, and the fluorescence was collected in the range above 530 nm. Image processing was carried out in the Image Lab™ Software and Fotor Photo Editor Software.

### 4.5. Flow Cytometry

Cells incubated with various agents for different times depending on the task were separated from the cultural flasks as described above. Next, the cells were transferred to test tubes, where complete medium was added in the ratio of cell suspension: complete medium = 1:3, centrifuged in an Eppendorf 5702 centrifuge for 5 min, 600 rpm, and the precipitate was dissolved in 1 mL of saline solution. Samples were analyzed on a CyFlow flow cytometer, Sysmex-Partec. For each sample, 50,000 events were recorded. To detect the fluorescence of the analyzed samples, a laser excitation at 488 nm was used collecting emission above 530 nm.

### 4.6. High Performance Liquid Chromatography and Mass Spectrometry

For the analysis of samples obtained by the method described above for TLC, reverse-phase high-performance liquid chromatography was used for product separation and mass spectrometry for more accurate identification of possible modifications of fluorescent dyes that occurred in the cell.

The Agilent 1260 instrument (Agilent Technologies, Santa Clara, CA) equipped with a PDA detector and a mass spectrometric detector, and a Zorbax SB-C8 column of 5 µm (150 mm × 4.6 mm; Agilent Technologies) with an acetonitrile gradient were used. In the experiments, electrospray ionization was employed. Only substances that fluoresce at wavelengths greater than 500 nm when excited with a wavelength of 488 nm were analyzed. The results were processed in MassLynx and ChemDoodle software.

### 4.7. Statistical Analysis

Statistical analysis was carried out in the Flowing Software 2.5.1 and Origin Pro 2016 software. The Wilcoxon test was used for quantitative comparison of two dependent samples (for example, cell fluorescence of the same culture, before and after the addition of any agents). The Mann-Whitney U-test was used to compare two independent samples, both astrocyte cultures and gliomas treated with the same agent. The significance level was set at 0.05.
